# Genomic Profiling of Circulating Tumor DNA From Cerebrospinal Fluid to Guide Clinical Decision Making for Patients With Primary and Metastatic Brain Tumors

**DOI:** 10.3389/fneur.2020.544680

**Published:** 2020-10-19

**Authors:** Lori A. Ramkissoon, Worthy Pegram, James Haberberger, Natalie Danziger, Glenn Lesser, Roy Strowd, Sonika Dahiya, Thomas J. Cummings, Wenya Linda Bi, Malak Abedalthagafi, Pratheesh Sathyan, Kimberly McGregor, Prasanth Reddy, Eric Severson, Erik Williams, Douglas Lin, Claire Edgerly, Richard S. P. Huang, Amanda Hemmerich, James Creeden, Charlotte Brown, Jeffrey Venstrom, Priti Hegde, Jeffrey S. Ross, Brian M. Alexander, Julia Elvin, Shakti H. Ramkissoon

**Affiliations:** ^1^Department of Pathology and Laboratory Medicine, University of North Carolina at Chapel Hill, Chapel Hill, NC, United States; ^2^Foundation Medicine, Inc., Morrisville, NC, United States; ^3^Foundation Medicine, Inc., Cambridge, MA, United States; ^4^Section of Medical Oncology and Hematology, Wake Forest Baptist Comprehensive Cancer Center, Winston-Salem, NC, United States; ^5^Department of Pathology and Immunology, Washington University School of Medicine, St. Louis, MI, United States; ^6^Department of Pathology, Duke University Medical Center, Durham, NC, United States; ^7^Department of Neurosurgery, Brigham and Women's Hospital, Boston, MA, United States; ^8^Genomics Research Department, Saudi Human Genome Project, King Fahad Medical City and King Abdulaziz City for Science and Technology, Riyadh, Saudi Arabia; ^9^Department of Pathology and Wake Forest Baptist Comprehensive Cancer Center, Wake Forest School of Medicine, Winston-Salem, NC, United States

**Keywords:** brain tumor, CSF (cerebrospinal fluid), genomic profiling, metastatic disease, circulating tumor DNA (ctDNA)

## Abstract

Despite advances in systemic therapies for solid tumors, the development of brain metastases remains a significant contributor to overall cancer mortality and requires improved methods for diagnosing and treating these lesions. Similarly, the prognosis for malignant primary brain tumors remains poor with little improvement in overall survival over the last several decades. In both primary and metastatic central nervous system (CNS) tumors, the challenge from a clinical perspective centers on detecting CNS dissemination early and understanding how CNS lesions differ from the primary tumor, in order to determine potential treatment strategies. Acquiring tissue from CNS tumors has historically been accomplished through invasive neurosurgical procedures, which restricts the number of patients to those who can safely undergo a surgical procedure, and for which such interventions will add meaningful value to the care of the patient. In this review we discuss the potential of analyzing cell free DNA shed from tumor cells that is contained within the cerebrospinal fluid (CSF) as a sensitive and minimally invasive method to detect and characterize primary and metastatic tumors in the CNS.

## Introduction

Comprehensive genomic profiling (CGP) of formalin-fixed paraffin-embedded (FFPE) tumor tissues has allowed many cancer patients to benefit from precision medicine by identifying genomic alterations for which targeted therapy or immunotherapy may be preferentially utilized. While the clinical value of tissue based CGP is well-established, this approach requires patients to undergo a tissue acquisition procedure such as a fine need aspiration, core biopsy, or surgical resection. As nearly all patients will be biopsied at presentation to establish a tissue diagnosis of malignancy, there is often tumor tissue available for CGP.

Despite advancements in therapeutic strategies for solid tumors, progression frequently occurs, manifesting as metastatic disease or recurrence. At this point, the ability to acquire a second biopsy can be challenging for several reasons including, the patient's general health status, the ability and/or willingness to undergo another invasive procedure, and the patient's safety associated with the location of the lesion to be biopsied. For patients with primary brain tumors (e.g., glioblastoma), most patients will undergo a tissue sampling procedure at the time of presentation to establish the diagnosis, while only a subset (10–20%) of patients will ever have a second procedure to acquire tissue at tumor recurrence (1, 2). Thus, 80% of primary brain tumor patients have no access to updated genomic data at the time of their post-initial treatment tumor progression highlighting a critical unmet need for this patient population. As such, in many instances when cancer patients are in critical need of new information to help guide their clinical management, tissue-based CGP may not be feasible.

To overcome this clinical challenge, recent advances in isolating circulating tumor DNA (ctDNA) from the plasma of peripheral blood samples has provided a novel mechanism for interrogating the genomic landscape of tumors without invasive surgical procedures. This type of assay is frequently referred to as a “liquid biopsy” and encompasses a broad category of minimally invasive tests that can effectively isolate DNA shed from cancer cells that are circulating in the blood or other relevant fluids. In contrast to circulating tumor cells (CTCs), which are intact tumor cells in the peripheral circulation, ctDNA is a component of the total circulating “cell-free” or cfDNA that includes both ctDNA and DNA fragments in plasma contributed by white blood cells and apoptotic normal non-hematologic cells. ctDNA testing platforms range from variant-specific and “hot-spot” assays to broad next generation sequencing panels that can assess for variants in hundreds of genes simultaneously. For some solid tumors, such as breast, lung, prostate, melanoma and colorectal cancers, ctDNA assays have demonstrated strong concordance of genomic profiles compared to tissue-based assays (3, 4). Moreover, ctDNA analysis for these patients before and after treatment have shown significant correlations between ctDNA levels and tumor response to treatment, highlighting further potential clinical utility for this platform both as stand-alone prognostic tests and potential monitoring assays.

While advanced-stage tumors demonstrate increasing yields of ctDNA in the plasma, detecting ctDNA in the plasma of primary brain tumor patients is limited, with only a minority yielding detectable levels. Across published reports, the rate of ctDNA detection in plasma for primary brain tumors varies from 10 to 50% of samples with higher detection rates associated with glioblastoma; however, all studies consistently report that even when detected, the ctDNA concentrations are much lower compared to other advanced stage tumors (3, 5–7). Although investigations are ongoing to determine why over half of high-grade glioma patients do not have detectable ctDNA in plasma, one likely contributor is the blood brain barrier (BBB) preventing both intact tumor cells and tumor cell-free DNA from reaching the peripheral circulation system. Recently one study demonstrated that the levels of plasma ctDNA from GBM patients were associated with increased BBB permeability, the density of macrophages around the tumor and the size of tumor vessels, supporting the hypothesis that the BBB integrity significantly influences ctDNA levels in the plasma of primary brain tumor patients (8).

Several studies have identified cerebrospinal fluid (CSF) as a rich and reliable source of ctDNA (ctDNA-CSF) in patients with primary and metastatic brain tumors. Secreted by the choroid plexus, CSF is a clear body fluid in the subarachnoid space of the brain and spinal cord that provides specialized immunologic protections and serves as a cushion for the brain (9). Given its direct contact with brain and relative ease of access through lumbar puncture, CSF could have major advantages over plasma for evaluating ctDNA for patients with metastatic and primary brain tumors.

In this review we will explore CSF testing for primary and metastatic brain tumors and discuss the clinical utility of testing for management of patients affected by brain cancer.

### Improved Yields From ctDNA-CSF Compared to Pelleted Cells From CSF

For tumors that arise outside of the CNS (e.g., lung cancer), the presence of tumor cells in the CSF correlates with poor response to therapy and overall poor prognosis (10, 11). To determine the spread of cancer to the brain or spinal cord, current methodologies rely on neuro-imaging, clinical findings such mental status changes, new onset headaches, presence of cauda equina syndrome, changes in sensory status and microscopic evaluation (cytology) or flow cytometry of CSF. For some cancers, particularly acute lymphoblastic leukemias, effective, early detection of infiltrating tumors cells in the CSF maximizes the ability to provide early and effective therapeutic intervention (12).

**Figure 1:**
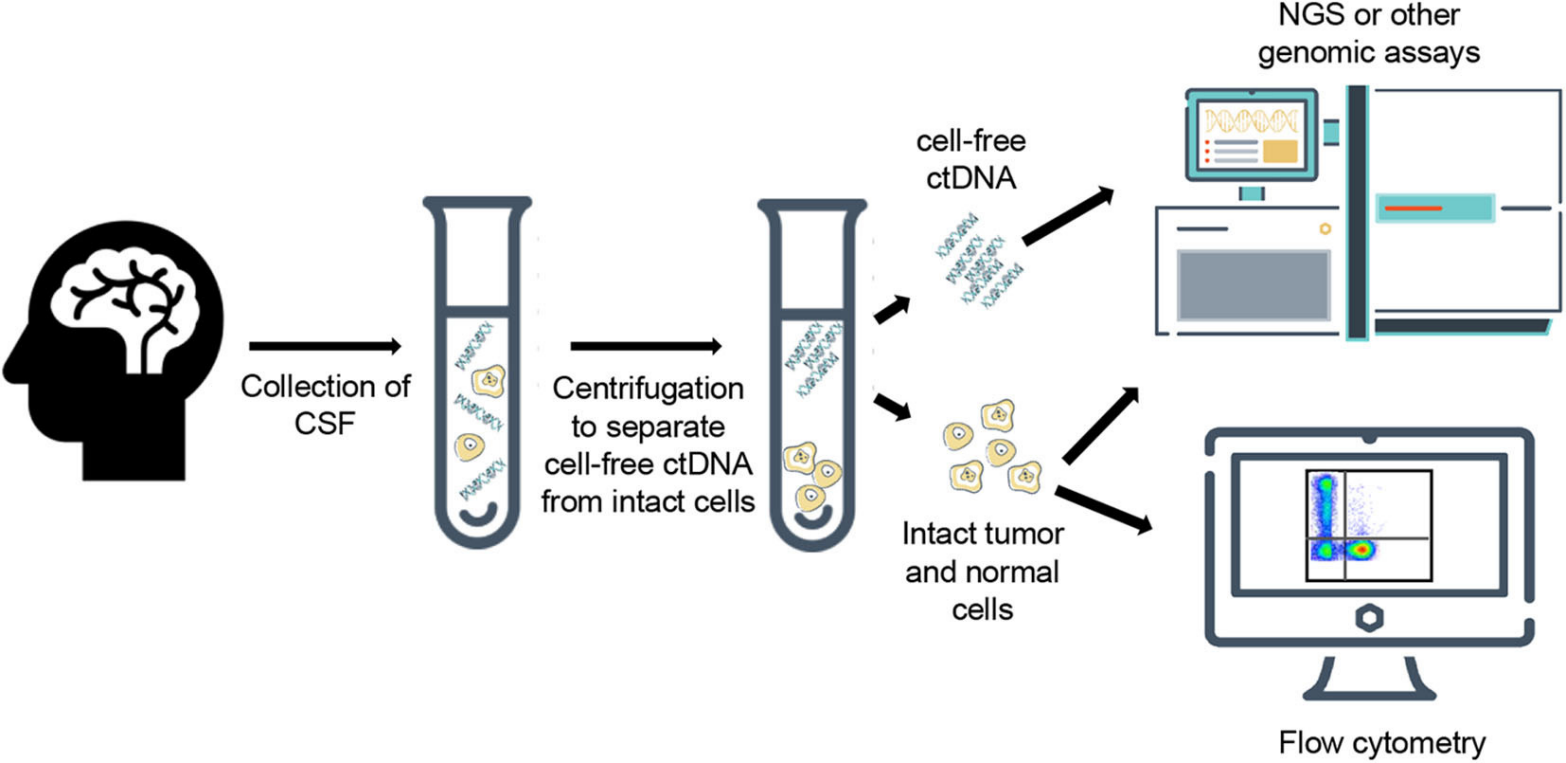
Generalized workflow for collection, processing, and analysis of cell-free ctDNA and intact cells from cerebrospinal fluid.

**Figure 2:**
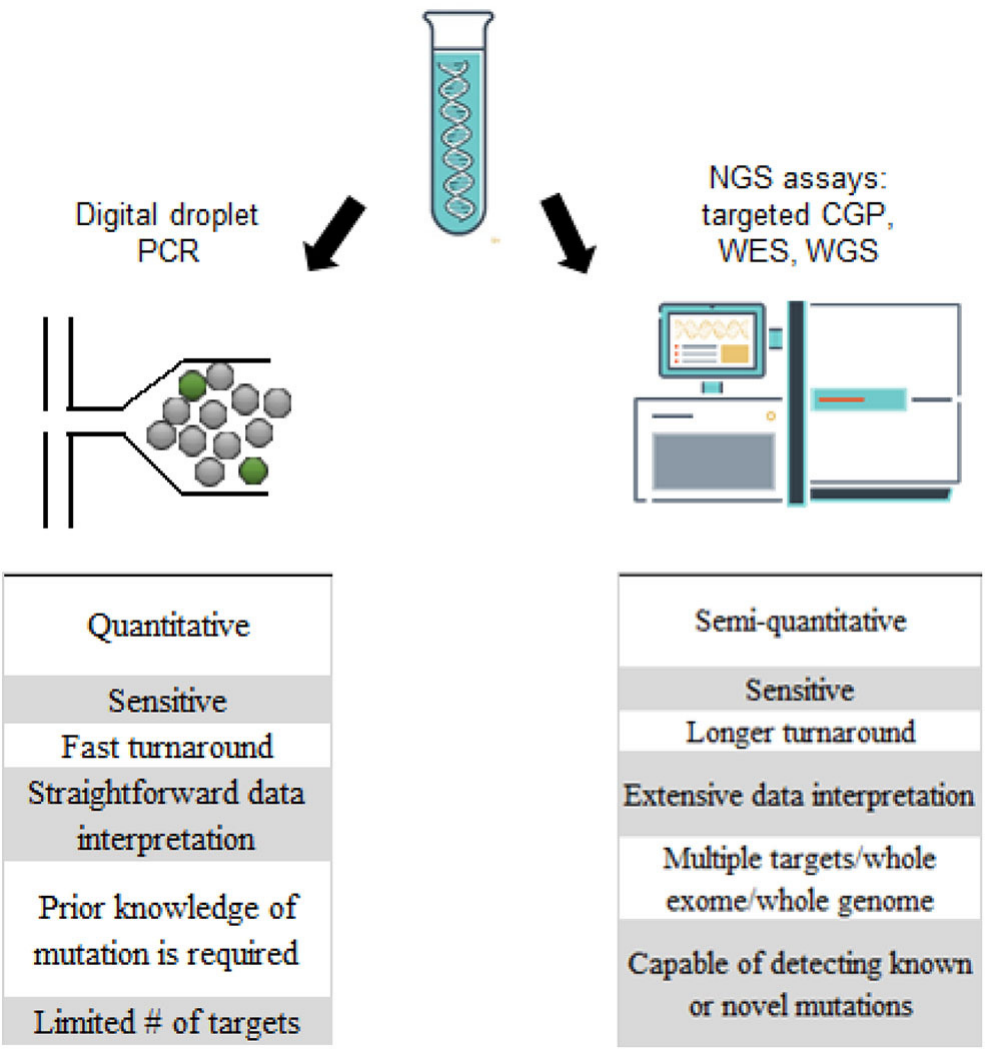
Comparison of digital droplet PCR (ddPCR) and next generation sequencing platforms in the analysis of ctDNA-CSF.

CSF is typically collected through a minimally invasive lumbar puncture but can also be accessed via existing shunts or during intracranial surgical procedures, with a typical volume of 2–10 mLs of fluid with each collection. Current methodologies for evaluating CSF focus on determining the presence of cancer cells with cytology, immunohistochemistry, or flow cytometry; however, while these techniques can be highly specific, they lack the sensitivity that is needed for early detection of malignancy. As an alternative, ctDNA from the CSF (ctDNA-CSF) can be extracted and sequenced, which increases sensitivity and provides additional information about tumor heterogeneity and progression of disease [(13); Figures 1, 2]. In the CSF, there are two sources of tumor DNA: intact cells and cell-free ctDNA, which is shed from tumor cells and circulates within the CSF. To collect the ctDNA-CSF, CSF samples are centrifuged within hours of collection and the supernatant carefully removed, while the remaining pellet can be re-suspended and used for additional analyses. Both the supernatant and cell pellet can either be frozen for later testing or the DNA can be immediately extracted. Specific kits for DNA extraction of circulating nucleic acids are available and typically protocols from the manufacturers are followed (14–16). The extracted DNA from both ctDNA-CSF in the supernatant and tumor cell DNA contained in the pelleted populations can then be evaluated for tumor-specific alterations, such as recurrent mutations, with more focused digital droplet PCR, or be evaluated with next-generation sequencing using a large gene panel, whole exome or genome platforms (Figures 1, 2).

Because normal white blood cells can be found within the CSF of both normal and cancer patients, several studies have been performed to compare enrichment of tumor specific variants in DNA from the CSF supernatant compared to DNA from pelleted cells within the CSF. Even though they are typically present in low numbers, the DNA obtained from white blood cells can potentially mask low-level tumor specific variants; therefore, determining which population provides more reliable, tumor-specific information is critical for achieving the greatest clinical utility. Pentsova et al. demonstrated in cancer patients with established brain metastases from solid tumors that although DNA yields were generally higher in the CSF pelleted population, a higher percentage of sequence reads with a known mutation was present in the ctDNA-CSF supernatant compared with the CSF pellet DNA (17). This trend was similar for copy number alterations as well, which was illustrated by a metastatic breast cancer sample that demonstrated significant *HER2* amplification in the ctDNA-CSF sample but barely detectable levels in the pelleted fraction. In all, known variants were detected in 100% of ctDNA-CSF samples but only 63% of CSF cell pellets from their cohort (17). Similarly, in a cohort of *EGFR*-mutated NSCLC patients, the *EGFR* mutation was detected in 100% (26/26) of ctDNA-CSF samples, however only 84.6% (22/26) and 73.1% (19/26) of pelleted CSF and plasma ctDNA samples, respectively, identified the mutation (4). The ctDNA-CSF allele fractions were also significantly higher compared to other sample types.

Importantly, multiple studies evaluating ctDNA-CSF in primary CNS tumors and brain metastases report high concordance between genomic profiles detected in tissue samples compared to those from ctDNA-CSF analyses (4, 17–19). Furthermore, there is strong evidence that ctDNA-CSF correlates with positive cytology results, wherein increasing levels of ctDNA-CSF are noted when cytology results are also positive for tumor cells. However, in patients with negative cytology findings but radiologic evidence for CNS metastases, ctDNA-CSF detected high-confidence somatic alterations in an average of 25% of patients (17). Another study reported that ctDNA-CSF detected mutations in 62% (5/8) patients with brain metastases that had negative cytology and radiologic findings for leptomeningeal disease (20). These studies and others highlight the increased sensitivity of ctDNA-CSF testing compared to traditional cytology studies (Table 1).

**Table 1:** Summary of studies demonstrating clinical utility of ctDNA-CSF in CNS disease.

**Study**	**Tumor types**	**Number of samples**	**Assay method**	**Analyte**	**Mutation detection rate**
De Mattos-Arruda et al. PMID: 26554728	Breast, lung, GBM	*n* = 12	Targeted sequencing	CSF	60%
				Plasma	55%
Pentsova et al. PMID: 27161972	Advanced solid tumors and primary CNS tumors	*n* = 32	Targeted sequencing	CSF	60–75%
Li et al. PMID: 29346604	NSCLC	*n* = 26	Targeted sequencing	CSF	100%
				Plasma	73%
Bettegowda et al. PMID: 24553385	Advanced solid tumors and primary CNS tumors	*n* = 640	Targeted sequencing	Plasma	>75% for most solid tumors
Zill et al. PMID: 29776953	Advanced solid tumors and primary CNS tumors	*n* = 21,807	Targeted sequencing	Plasma	51–93%
Wang et al. PMID: 26195750	Primary CNS tumors	*n* = 35	Targeted sequencing	CSF	74%
Miller et al. PMID: 30675060	Primary CNS tumors	*n* = 85	Targeted sequencing	CSF	50%
				Plasma	0%
von Baumgarten et al. PMID: 31903155	Advanced solid tumors	n=27	Targeted sequencing	CSF	74%
Martinez-Ricarte et al. PMID: 29615461	Primary CNS tumors	*n* = 20	Targeted sequencing	CSF	79%
Momtaz et al. PMID: 27863426	Advanced melanoma	*n* = 11	Digital droplet PCR (ddPCR)	CSF	55%
Schwaederle et al. PMID: 26848768	Advanced solid tumors and GBM	*n* = 171	Targeted Sequencing	Plasma	65%
Piccioni et al. PMID: 30855176	Primary CNS tumors	*n* = 419	Targeted sequencing	Plasma	50%
Mouliere et al. PMID: 30401727	Primary CNS tumors	*n* = 13	Shallow whole genome sequencing	CSF	39%
				Plasma	
Pan et al. PMID: 25605683	Advanced solid tumors and primary CNS tumors	*n* = 7	ddPCR	CSF	86%
			Targeted sequencing	Plasma	
Bobillo et al. PMID: 32079701	B-cell lymphoma	*n* = 19	ddPCR	CSF	32%
			Targeted sequencing	Plasma	
Huang et al. PMID: 31161597	Lung adenocarcinoma	*n* = 35	ddPCR	CSF	50–75%
				Plasma	

### Clinical Utility of ctDNA-CSF Testing in CNS Metastases

The development of metastatic disease involving the CNS is associated with a poor prognosis, therefore, a first step to improving outcomes for these patients is understanding the genomic landscape of brain metastases which most often go unsampled. Testing of ctDNA-CSF may also help confirm CNS involvement in instances where the results of neuro-imaging studies are equivocal. Current methodologies to detect tumor metastases or neoplastic meningitis rely on radiologic changes, correlation of pertinent clinical symptoms and/or the presence of tumor cells in the CSF by cytology; however, these methods lack the sensitivity required to identify disease early, necessitating the need for more sensitive, minimally invasive approaches.

For non-CNS solid tumors there is growing evidence that monitoring ctDNA from plasma provides information about systemic disease response to treatment and emergence of targeted therapy-associated resistance mutations. Because of the strong concordance of mutational profiles between tissue and ctDNA, monitoring the levels and mutational status of ctDNA in breast, colorectal and non-small cell lung cancers have demonstrated significant clinical utility. Indeed, in breast cancer patients increasing ctDNA levels are associated with inferior survival and increased risk of recurrence, while one study reported 96% of *EGFR*-mutated colorectal or NSCLCs demonstrated a reduction in ctDNA plasma levels after initiation of targeted therapy (21).

It has been shown that in more than 50% of cases, solid tumor metastases to the brain harbor clinically relevant mutations that were not detected in the primary tumor (22). Monitoring mutational profiles of tumors with a high rate of metastasis over the course of a patient's treatment plan can provide meaningful information that may guide clinical management. Importantly for brain metastases, several studies have demonstrated that analyzing ctDNA-CSF offers a more sensitive and specific source of tumor-specific mutations compared to plasma ctDNA (4, 19, 23). In one study from a cohort of 53 patients with molecular profiling of ctDNA-CSF, 12 patients developed CNS tumor progression while on a targeted kinase inhibitor therapy. Of these patients, ctDNA-CSF analysis detected a drug-resistance mutation in 33% of patients. Importantly these mutations were not identified in the primary tumor tissue (17). Similarly, in a cohort of 26 *EGFR*-mutated NSCLC patients with leptomeningeal metastases, unique genomic profiles were detected from ctDNA-CSF compared to the primary tumor or plasma ctDNA, including increased *MET* copy number gains and *TP53* loss of heterozygosity (4). Additionally, it has been shown that ctDNA-CSF harbors private mutations that are restricted to the metastatic brain lesions and could not be identified in other metastatic sites present in the patient. In one Li-Fraumeni patient with both HER2-positive metastatic breast cancer and esthesioneuroblastoma, the authors demonstrated that ctDNA-CSF mutation profiles were specific to the brain metastasis, while plasma ctDNA testing reported mutations found only in the extracranial esthesioneuroblastoma (19).

Of great clinical significance, *EGFR* resistance mutations can often be detected in plasma from NSCLC patients prior to any clinical signs of progression, suggesting that monitoring ctDNA levels and mutational profiles over the disease course could provide opportunities for earlier therapeutic intervention. The *EGFR* resistance mutation T790M was also detected more frequently in ctDNA-CSF (30%) compared to plasma ctDNA (21%). In another study of patients with leptomeningeal carcinomatosis, ctDNA-CSF analysis detected the presence of disease in 100% of cases while neuroimaging and CSF cytology identified 63 and 71% of cases, respectively. In another institution's experience, ctDNA-CSF genomic profiling from patients with malignant CNS disease detected clinically actionable mutations in 40% (11/27) of patients. After consultation with an interdisciplinary molecular tumor board, seven patients were recommended to receive a targeted therapy based on the ctDNA-CSF results, with four patients being clinically stable to proceed with the new treatment regimen. These patients included two metastatic NSCLC patients with *EGFR* mutations who received erlotinib or afatinib, a NSCLC patient with an *EZR/ROS1* fusion who was treated with crizotinib and a HER2 positive metastatic breast cancer patient with *FGFR* amplification who received everolimus (20). This study highlights the potential clinical applications for ctDNA-CSF testing in CNS disease, wherein identifying targetable alterations in brain metastases may offer new therapeutic opportunities.

### Clinical Utility of ctDNA-CSF Genomic Profiling in Gliomas

While ctDNA from plasma provides a reliable, representative source for establishing and monitoring mutational profiles for extra-cranial solid tumors, it does not offer the same sensitivity for primary brain tumors. Bettegowda et al. demonstrated that ctDNA from plasma is detectable in the vast majority of patients with advanced cancers such as pancreatic, ovarian, colorectal, breast, melanoma, and hepatocellular tumors, but <10% of glioma patients had measurable levels of ctDNA (3). In their study of 136 metastatic tumors, ctDNA was detected in 82% (112/136) of cases, however the levels the ctDNA differed significantly between tumor types. For primary brain tumors, half of the medulloblastoma cases had detectable ctDNA in plasma (7/14), while only two glioma patients showed detectable levels (2/27). Similarly, another study of 33 GBMs showed that 73% of tumors did not have any detectable ctDNA in plasma (7). In a separate report, de Mattos et al. demonstrated that glioma specific mutations were not detected in plasma from any of the patients tested, which likely reflects the restrictive nature of the BBB to prevent glioma cells and ctDNA from leaving the CNS. In this study the sensitivity of ctDNA from plasma was compared to CSF and in all four primary glioma samples, ctDNA-CSF detected mutations that were identified in the primary tissue, while these variants were not detected in plasma ctDNA.

Liquid biopsies of the CSF have been explored as a less invasive method for obtaining molecular information specific to the tumor that can aid in the diagnosis, prognosis, and treatment for brain tumor patients. Several studies have demonstrated that ctDNA from primary brain tumors is enriched in CSF, with detection rates similar to those reported for other advanced cancers using ctDNA from plasma (17, 19, 24). The ability to detect ctDNA in the CSF does not appear to correlate with patient demographics, presence of hydrocephalus, contrast enhancement or tumor size; however, the levels of ctDNA-CSF in one study correlated with tumor grade, with higher levels of ctDNA found in high-grade tumors compared to low-grade lesions (25). This correlation, however, did not hold true for adult gliomas as a larger study that analyzed 85 WHO grade II-IV adult gliomas did not find an association between ctDNA-CSF positivity and tumor grade (24). The strongest factor influencing ctDNA-CSF levels, however, is anatomic location. When tumors are adjacent to CSF spaces, such as cortical surfaces and ventricles, a higher percentage of tumors have detectable ctDNA levels compared to tumors that are encapsulated (24, 25). In a study of 20 diffuse gliomas, only three cases were identified wherein mutational profiles from ctDNA-CSF did not match the mutations detected in tissue, while the other 17 samples had corresponding genomic profiles in the CSF and the primary tumor. It was hypothesized that the distance from the cortex or ventricles contributed to the discordance (26).

The molecular characterization of the ctDNA-CSF in patients with primary brain tumors has demonstrated the ability to capture a broad spectrum of mutations and copy number alterations that resemble the mutational profiles of tumor biopsies. In adult gliomas, comparison of the tumor mutation burden and frequency of mutations was comparable between ctDNA-CSF and tissue genomic analyses. The most commonly detected alterations in ctDNA-CSF were truncal variants including *TERT* promoter, *TP53* and *IDH1* mutations as well as *CDKN2A/B* deletions. Clonal mutations are more likely to also be detected in ctDNA-CSF, however, subclonal mutations were more likely to be identified in tissue (24, 27).

Longitudinal studies have also highlighted the ability of ctDNA-CSF to capture the genomic response to treatment and tumor evolution. In one patient with a 1p/19q co-deleted oligodendroglioma, ctDNA-CSF analysis 7.5 years after the initial diagnosis reported >400 non-synonymous single nucleotide variants in a pattern commonly associated with exposure to alkylating agents such as temozolomide (17). Additionally, when CSF samples collected at diagnosis were compared to CSF collected at the time of recurrence (18 months) the ctDNA-CSF mutational profiles diverged with later samples harboring an increase in mutations of genes associated with growth factor signaling pathways or in genes that were mutated in the primary sample (24). The molecular evolution observed from ctDNA-CSF analyses recapitulated the patterns that have been previously reported in studies of sequential tumor biopsies, highlighting the possibility that ctDNA-CSF could substitute for tissue testing in some patients.

Published studies have demonstrated that detection of ctDNA-CSF provides prognostic implications for glioma patients. The presence of ctDNA-CSF was associated with a four-fold higher risk of death in adult glioma patients compared to patients without detectable levels. To illustrate the increased sensitivity of ctDNA-CSF, most of the patients with ctDNA-positive CSF did not have detectable malignant cells in the CSF by cytopathologic analysis. In a multivariate analysis, the presence of ctDNA-CSF remained a statistically significant prognostic factor (24).

### Clinical Utility in Non-glial Primary CNS Tumors

The utility of molecular profiling of ctDNA-CSF is not limited to primary glial tumors as other CNS tumors face similar diagnostic and clinical management challenges. Exploring the sensitivity and specificity of ctDNA-CSF in non-glial CNS tumors may provide alternative methods that can aid in diagnosis or identification of disease recurrence.

Although primary germ cell tumors (GCTs) only represent 1% of primary brain tumors in pediatric and young adult patients, the CNS is the second most common location for extragonadal tumors. These are a heterogeneous group of tumors that can be difficult to diagnose because of their non-specific clinical presentations and subsequently predict which tumors are more likely to metastasize to the brain. The propensity to spread throughout the CNS increases the need for highly specific and sensitive markers that can be detected in the CSF of patients. Currently monitoring certain protein biomarkers such as alpha-fetoprotein and human chorionic gonadotropin within the CSF only provides information for certain GCT subtypes and has limited sensitivity in terms of diagnosing CNS dissemination (28). There is however increased interest in evaluating the utility of molecular biomarkers present in the CSF of GCT patients. One study has demonstrated the feasibility to detect microRNAs in CSF that have been reported to be overexpressed in malignant germ cell tissues (29).

Primary CNS lymphoma (PCNSL) patients may also benefit from CSF liquid biopsies as these tumors often disseminate via CSF. Notably, surgical resection is not standard of care, and if PCNSL is diagnosed during an intraoperative frozen section, the surgical procedure is routinely stopped as this disease is best managed with chemotherapy. While this is considered best practice, it does limit the amount of tissue available for genomic testing and further reduces the likelihood of additional tissue sampling at the time of tumor recurrence. Detection of mutational profiles and immunoglobulin gene rearrangements in CSF has the potential to improve diagnostic sensitivity, especially in setting of steroid administration, which can make tissue sampling of PCNSL challenging because of tumor cell lysis. In contrast, one can speculate that the use of steroids for PCNSL prior to ctDNA-CSF liquid biopsy may enhance the amount of ctDNA available in the CSF compartment. In one study 19 patients with B-cell lymphomas were evaluated to compare the utility of ctDNA-CSF or plasma in diagnosing CNS involvement and explore whether the analysis of ctDNA could monitor treatment response in CNS lymphomas (30). Genomic analysis of ctDNA from either CSF or plasma demonstrated readily detectable ctDNA-CSF in all 6 cases of CNS restricted lymphomas, while plasma ctDNA was only detected in 2 out of 6 cases, highlighting the sensitivity of ctDNA-CSF compared to plasma. This analysis also showed that tumor burden, measured by MRI or flow cytometry, was concordant with ctDNA-CSF levels but not plasma ctDNA. Moreover, ctDNA-CSF demonstrated an increased sensitivity for detecting CNS involvement compared to conventional cytology or flow cytometry (30).

Further utility for CSF liquid biopsy in PCNSL includes identifying tumor recurrence, particularly in cases associated with extensive leptomeningeal involvement in which diagnosing recurrent tumor in the post-treatment setting solely by neuro-imaging studies can be very difficult, often when cytologic analysis is unrevealing. Indeed, sequential ctDNA-CSF testing in patients undergoing treatment demonstrated that despite a decrease in tumor cells by flow cytometry or in some cases no detectable disease by conventional techniques, patients still harbored CNS disease when assessed with a more sensitive method (30).

With recent data highlighting clinical utility of the Bruton tyrosine kinase inhibitor (BTKi) ibrutinib as monotherapy in salvage treatment of PCNSL, this highlights the value of mapping the genomic landscape of recurrent PCNSL. While BTKi may become an important option for salvage therapy, it is worth noting that several mechanisms of resistance have been identified including mutations in *MYD88, CARD11, TNFAIP3*, and *CD79B*. Therefore, in the recurrent PCNSL setting, should CSF liquid biopsy identify mutations in these key genes, it would likely guide therapy away from BTKi (31–33).

### Clinical Decision Making for Brain Tumor Patients Based on ctDNA-CSF Testing

Liquid biopsies are increasingly demonstrating clinical utility for a variety of cancers. Guidelines have evolved most rapidly in non-CNS cancers such as lung cancer where CAP guidelines suggest that liquid biopsy may be acceptable at the time of diagnosis should tissue not be available for testing.

Additionally, this methodology can help clarify radiologic findings and differentiate between radiation-induced necrosis and tumor progression. Pseudoprogression, defined as post-radiotherapy changes that typically resolve spontaneously, occurs in 10–30% of GBM patients typically within the first 12 weeks of treatment (34). The ability to distinguish these changes from true disease progression is critical for patient management, as deciding between the scenarios can mean additional treatment, change in treatment strategy, or another invasive surgical intervention. Currently there is a need for specific biomarkers that can be used to discriminate between disease recurrence and pseudoprogression. cfDNA has demonstrated utility in discerning between these in non-CNS tumors and initial studies are providing proof of concept evidence that the presence of tumor specific ctDNA yield (ng) and mutations in ctDNA-CSF after treatment are biomarkers that can be used in conjunction with radiologic findings to guide clinical management (35–37).

Molecular assessment of ctDNA-CSF can also assist in diagnosis and classification of tumors which, due to their anatomic location in the CNS, cannot be biopsied for a histologic diagnosis. For example, in patients with midline gliomas such as diffuse intrinsic pontine glioma (DIPG), given the anatomic location, it is often not possible to biopsy these patients and therefore they are routinely treated clinically based on imaging studies. If however ctDNA-CSF testing was available and showed a *H3F3A* (K27M) mutation, it would suggest a molecular diagnosis of diffuse midline glioma, *H3F3A* K27M mutant, WHO Grade IV; alternatively, if the tumor harbored a *KIAA1549-BRAF* fusion, that would suggest a lower grade glioma such as pilocytic astrocytoma WHO Grade I. A retrospective study of 20 glioma patients demonstrated combined digital droplet PCR and targeted sequencing of known glioma associated mutations on ctDNA-CSF samples could correctly classify gliomas in 85% of cases; the only samples that failed were low-grade gliomas or tumors where ctDNA-CSF yields were low (26). Prospective studies are needed to define the sensitivity, specificity, and reproducibility of ctDNA-CSF for diagnosing gliomas.

There is significant potential for ctDNA-CSF testing to disrupt the way neuro-oncology is currently practiced by providing neurosurgeons, oncologists and patients with pre-surgical, pre-radiation and pre-systemic therapy genomic diagnoses that can inform clinical management strategies. The value of total and near-total resection for high grade gliomas remains an important therapeutic and prognostic indicator. ctDNA-CSF molecular analysis has the potential to provide more information to neurosurgeons for planning surgical procedures based on genomic profiles. In instances where the tumor is based in an eloquent area of the brain or deep gray structures where resection is not possible and the purpose of surgical intervention is solely to sample tissue for diagnosis, CSF ctDNA testing may be an alternative to biopsy procedures.

As several studies have demonstrated, ctDNA-CSF sequencing can also provide opportunities for early detection of metastatic disease, which may have significant implications for therapeutic intervention. Retrospective studies have demonstrated that in the context of metastatic disease ctDNA-CSF mutations reflect CNS specific alterations, which are not identified in plasma ctDNA or extracranial metastases (19). Incorporating ctDNA-CSF genomic analysis along with traditional cytology, radiologic and other methodologies will refine the utility and sensitivity of detecting CNS disease. Early detection of novel activating mutations in ctDNA-CSF provides the opportunity to investigate utility of targeted therapies, while detection of resistance mutations offers the ability to treat with combination therapies that target multiple primary therapy resistant clones. Furthermore, monitoring levels of ctDNA-CSF can offer insights into the efficacy of therapeutic strategies and potentially guide dosing and treatment duration. For HER2 positive breast cancer patients, treatment with monoclonal antibodies or tyrosine kinase inhibitors demonstrate significant efficacy for systemic disease, however these therapies also increase the prevalence of CNS metastases as the brain may protect tumor cells from the effects of these treatments (38, 39). ctDNA-CSF has shown significant utility in providing biomarkers to demonstrate response to treatment for CNS disease. One study demonstrated that in contrast to plasma ctDNA which showed decreasing *ERBB2* levels and other tumor-specific mutations during treatment with trastuzumab emtansine, CSF ctDNA revealed continued high levels of *ERBB2, MYC* copy numbers, *PIK3CA*, and *TP53* mutations, which were identified prior to the start of treatment (40). This highlights the ability of ctDNA-CSF monitoring to inform how metastatic CNS disease is responding to treatment. In one study monitoring *H3F3A* K27M levels in CSF ctDNA correlated with the presence of contrast-enhancing areas on MRI in pediatric DIPGs (41).

Importantly, to fully understand the potential of ctDNA-CSF testing and incorporate the methodology into routine clinical care, prospective clinical trials are needed. Larger studies should be performed that evaluate the correlations between ctDNA-CSF, plasma ctDNA and tumor tissue samples. Future prospective studies that incorporate ctDNA-CSF testing in parallel with standard of care diagnostic and management procedures will help refine and implement the utility of ctDNA-CSF testing for patients with brain metastases and primary tumors.

## Author Contributions

All of the authors contributed to the preparation and writing manuscript as each individual has specific expertise in different areas, including neuro-oncology, metastatic CNS disease, circulating tumor DNA, genomic profiling, neurosurgery, and integration of molecular data into direct clinical care.

## Conflict of Interest

Some authors are employed by Foundation Medicine, Inc. The authors declare that the research was conducted in the absence of any commercial or financial relationships that could be construed as a potential conflict of interest.
